# Implementing international sexual counselling guidelines in hospital cardiac rehabilitation: development of the CHARMS intervention using the Behaviour Change Wheel

**DOI:** 10.1186/s13012-016-0493-4

**Published:** 2016-10-10

**Authors:** J. Mc Sharry, P. J. Murphy, M. Byrne

**Affiliations:** Health Behaviour Change Research Group, School of Psychology, NUI Galway, Galway, Ireland

**Keywords:** Behaviour change, Intervention development, Complex intervention, Implementation, Cardiac rehabilitation, Cardiovascular disease, Sexual counselling

## Abstract

**Background:**

Decreased sexual activity and sexual problems are common among people with cardiovascular disease, negatively impacting relationship satisfaction and quality of life. International guidelines recommend routine delivery of sexual counselling to cardiac patients. The Cardiac Health and Relationship Management and Sexuality (CHARMS) baseline study in Ireland found, similar to international findings, limited implementation of sexual counselling guidelines in practice. The aim of the current study was to develop the CHARMS multi-level intervention to increase delivery of sexual counselling by healthcare professionals. We describe the methods used to develop the CHARMS intervention following the three phases of the Behaviour Change Wheel approach: understand the behaviour, identify intervention options, and identify content and implementation options. Survey (*n* = 60) and focus group (*n* = 14) data from two previous studies exploring why sexual counselling is not currently being delivered were coded by two members of the research team to understand staff’s capability, opportunity, and motivation to engage in the behaviour. All potentially relevant intervention functions to change behaviour were identified and the APEASE (affordability, practicability, effectiveness, acceptability, side effects and equity) criteria were used to select the most appropriate. The APEASE criteria were then used to choose between all behaviour change techniques (BCTs) potentially relevant to the identified functions, and these BCTs were translated into intervention content. The Template for Intervention Description and Replication (TIDieR) checklist was used to specify details of the intervention including the who, what, how and where of proposed intervention delivery.

**Results:**

Providing sexual counselling group sessions by cardiac rehabilitation staff to patients during phase III cardiac rehabilitation was identified as the target behaviour. Education, enablement, modelling, persuasion and training were selected as appropriate intervention functions. Twelve BCTs, linked to intervention functions, were identified for inclusion and translated into CHARMS intervention content.

**Conclusions:**

This paper details the use of Behaviour Change Wheel approach to develop an implementation intervention in an under-researched area of healthcare provision. The systematic and transparent development of the CHARMS intervention will facilitate the evaluation of intervention effectiveness and future replication and contribute to the advancement of a cumulative science of implementation intervention design.

**Electronic supplementary material:**

The online version of this article (doi:10.1186/s13012-016-0493-4) contains supplementary material, which is available to authorized users.

## Background

The gap between *what we know* and *what we do*, between research evidence and clinical practice, is a consistent feature of healthcare delivery [1, 2]. Changing healthcare professional behaviour to implement evidence-based guidelines into routine practice is a major challenge within the time and resource constraints of the healthcare system. Implementing guidelines for sensitive topics, such as sexuality, can be particularly difficult with healthcare professionals’ discomfort and fear of causing offence acting as additional barriers to changing patient care [3]. Obstacles to implementation can arise at multiple levels within the healthcare system: from the individual patient to the wider environment [4].

Multi-level interventions are interventions targeted at several levels, for example, individual patients, groups, and organisations [5]. Implementation interventions, to improve the update of evidence into practice, can be understood as multi-level interventions that require patient, provider, system and environmental level change [6]. The overall goal of implementation interventions is to enhance care at a *patient level* to improve patient outcomes. Implementing evidence into practice often requires intervention at the *provider level* to support healthcare professionals to modify established patterns of care. Ensuring sustainability requires interventions to be integrated at a *system level* and to work within healthcare infrastructures for adoption into routine care. Finally, at an *environmental level*, the widespread adoption of new practices requires the development of policy frameworks that embed implementation at a national or international level.

Recent years have seen advancements in behavioural science of particular relevance to the healthcare professional behaviour change at the heart of provider level interventions [7]. Systematic guidance and lists of behavioural techniques provide tools to develop theory-based behaviour change interventions, detail the mechanisms through which change is expected to occur and describe intervention content using shared terminology [8, 9]. The current study explores the use of these tools and techniques to develop a multi-level implementation intervention to promote the provision of sexual counselling to cardiac patients.

Sexual counselling is an under-researched aspect of healthcare provision. Sexual dysfunction among patients with cardiovascular disease is common, and return to sexual activity after a cardiac event is challenging [10, 11]. Sexual problems can have far-reaching and negative consequences on psychological well-being, relationship satisfaction and quality of life [12, 13]. There is evidence that sexual counselling can reduce sexual problems and improve sexual function [14]. The World Health Organization has rated the evidence that brief sexuality-related counselling is more effective than usual care in reducing sexual difficulties as strong [15]. Sexual counselling for cardiac patients aims to assess existing sexual problems, provide information on concerns and support safe return to sexual activity after a cardiac event or procedure [16]. A consensus document endorsed by the American Heart Association and European Society of Cardiology recommends routine assessment of sexual problems and sexual counselling for all individuals with cardiovascular disease [17, 18]. Despite consensus guidelines, and evidence of patient need, discussions between patients and providers about sexual activity rarely take place in practice [19].

Exploring the healthcare context is recommended for the effective implementation of guidelines into practice [6]. In Ireland, the Cardiac Health and Relationship Management and Sexuality (CHARMS) baseline study surveyed nationally representative samples of patients, general practitioners and cardiac rehabilitation staff about sex and cardiovascular disease [20]. In line with international evidence, Irish cardiac patients reported high rates of sexual problems; nearly double the rate for age-matched non-cardiac samples [21]. Patients recognised the need for support, with the majority interested in receiving sexual education and counselling. This need was not being met; two thirds of patients reported that sex had never been addressed by their healthcare provider, and when addressed, satisfaction with the manner of provision was low [21]. General practitioners reported rarely discussing sexual concerns with cardiac patients [22], and cardiac rehabilitation staff reported a lack of specific assessment and counselling guidance within their service [23].

We aimed to develop the CHARMS complex multi-level intervention to address the poor implementation of evidence-based guidelines to provide sexual counselling to cardiac patients in Ireland. The overall aims of the CHARMS intervention were to:

- Improve sexuality-related outcomes for patients with cardiac disease (patient level)
- Increase provision of sexual counselling by healthcare providers (provider level)
- Develop a sustainable pathway for the delivery of sexual counselling within the Irish healthcare system (system level)

As there are very few examples of documented sexual counselling interventions and existing examples are poorly described [24], we chose to take a systematic approach to intervention development and description. The Behaviour Change Wheel (BCW) is a framework for designing interventions developed by synthesising 19 existing behaviour change frameworks [8]. Step-by-step guidance on the use of the BCW in the systematic design of interventions has been outlined and linked to the UK Medical Research Council (MRC) guidance for the development of complex interventions [25, 26]. At the centre of the BCW is the Capability Opportunity Motivation-Behaviour (COM-B) model, a means to understand behaviour in context. Understanding the behaviour of health professionals in context facilitates the development of interventions to target barriers to change that behaviour. Tailoring interventions to overcome context-specific barriers has been shown to improve care delivery and patient outcomes [27].

The COM-B model describes behaviour as an interaction between an individual’s capability, opportunity and motivation to engage in the behaviour. Capability is the individual’s ability to perform a behaviour and includes both physical capability (e.g. skills) and psychological capability (e.g. knowledge). Opportunity describes the factors that lie outside the individual that facilitate or prompt behaviour and includes both physical opportunity (e.g. affordability) and social opportunity (e.g. cultural norms). Motivation describes the brain processes that energise and direct behaviour and includes both automatic motivation (e.g. habits) and reflective motivation (e.g. cost-benefit decision making). The BCW links COM-B components to nine intervention functions (coercion, education, enablement, environmental restructuring, incentivisation, modelling, persuasion, restriction, training), through which an intervention can change behaviour, and seven broad policy categories (communication/marketing, environmental/social planning, fiscal measures, guidelines, legislation, regulation, service provision) describing the decisions authorities can make to support delivery of intervention functions [28].

The BCW approach links intervention functions to behaviour change techniques (BCTs), the observable, replicable and irreducible active ingredients of an intervention, outlined in the BCT Taxonomy v1 (BCTTv1), a structured list of BCTs [29]. The agreed labels and definitions included in the BCTTv1 allow intervention content to be clearly described using standard terminology [9, 29]. Existing studies have used the BCW, the COM-B model and BCT taxonomies to develop and describe implementation interventions in healthcare settings [26, 30, 31].

This paper describes the methods used to develop the CHARMS intervention to increase the provision of sexual counselling to cardiac rehabilitation patients in Ireland following the BCW approach. By describing the use of the BCW to implement international guidelines in practice, we hope to provide guidance for future implementation intervention designers and to reflect on the usefulness of the BCW in the development of implementation interventions. The main target of behaviour change was at the provider level, to increase the provision of sexual counselling by healthcare professionals. However, as changing provider behaviour requires patient level interaction and system level integration, the multi-level nature of the intervention was considered throughout development.

## Methods

We used the BCW to guide intervention development. BCW guidance provides a step-by-step method for designing behaviour change interventions and outlines three phases in the intervention design process: understand the behaviour, identify intervention options and identify content and intervention options [28].

### Phase 1: Understand the behaviour

Phase 1 describes four steps that lay the ground work for understanding the target behaviour: define the problem in behavioural terms, select the target behaviour, specify the target behaviour and identify what needs to change.

#### Step 1: Define the problem in behavioural terms

Step 1 requires describing the problem of interest as a behaviour and specifying the target group involved and the behaviour itself. We used guideline recommendations [17, 18] and documented evidence-practice gaps in Ireland [21, 23, 32] to define the problem in behavioural terms.

#### Step 2: Select target behaviour

Step 2 describes generating a long list of potential candidate behaviours and selecting a target behaviour by considering each behaviour in terms of potential impact, likelihood of change, spillover effect and ease of measurement. We did not engage with this step in the process, as the target behaviour was pre-determined from international guidelines.

#### Step 3: Specify the target behaviour

We followed BCW guidance to specify the target behaviour in appropriate detail and in context by answering the following questions: What is the behaviour that will be targeted for change? Who performs the behaviour? When and where do they perform the behaviour? In answering these questions to specify provider level behaviour change, we maintained a system level awareness of the reality of Irish healthcare. We answered these questions with reference to published recommendations on the delivery of sexual counselling to individuals with cardiovascular disease [17, 18]. We identified the setting for our implementation intervention through a system-level consideration of the most sustainable method of delivery within Irish healthcare and by incorporating the views of patients, general practitioners and cardiac rehabilitation staff elicited during the CHARMS baseline studies [32].

#### Step 4: Identify what needs to change

We used two of the CHARMS baseline studies to understand why the target behaviour was not currently being carried out and to identify what needs to change: a nationally representative quantitative survey of cardiac rehabilitation staff (*n* = 60) views of sexual counselling [23] and a qualitative study of staff (*n* = 14) experiences of discussing sexual issues with patients [32]. From the quantitative survey, endorsed attitudes and beliefs and perceived barriers to the provision of sexual counselling were extracted, as were self-ratings of knowledge, confidence and awareness in relation to sexual counselling. From the qualitative study, reported barriers from focus group data were extracted. Data from both studies were then coded to COM-B model components by PM and JMS.

### Phase 2: Identify intervention options

Phase 2 describes two steps to guide selection of intervention options: identify intervention functions, the broad categories through which an intervention can change behaviour, and identify policy categories to support the delivery of the identified functions.

#### Step 5: Identify intervention functions

We identified potentially relevant intervention functions by linking the COM-B components relevant to why sexual counselling is not currently being carried out, with intervention functions as per published guidance on the use of the BCW [8]. We then used the APEASE (affordability, practicability, effectiveness and cost effectiveness, acceptability, side effects/safety and equity) criteria to select the most appropriate intervention functions for our intervention [28].

#### Step 6: Identify policy categories

BCW guidance suggests which policy categories are likely to be appropriate and effective in supporting different intervention functions. For each intervention function identified in step 5, we listed the policy categories suggested in BCW guidance. We then identified the policy categories common across our included intervention functions and selected those we believed to be most applicable to the cardiac rehabilitation setting [28].

### Phase 3: Identify content and implementation options

Phase 3 describes two steps to specify intervention content in more detail in terms of BCTs and to identify the mode of delivery for the intervention.

#### Step 7: Identify behaviour change techniques

We used the labels and detailed definitions included in the BCTTv1 [9] to identify BCTs. We took the intervention functions identified during step 5 and prepared an initial long list of potential BCTs using published guidance mapping BCTs to intervention functions [28]. BCTs that did not meet the APEASE criteria within the context of cardiac rehabilitation were excluded, and the most appropriate BCTs were shortlisted by JMS, PM and MB separately. We decided on final BCT inclusion through group discussion.

#### Step 8: Identify mode of delivery

JMS, PM and MB first separately brainstormed the best ways to translate BCT into intervention content and then met to discuss and agree on content. We used the Template for Intervention Description and Replication (TIDieR) checklist to specify details of the intervention including the who, what, how and where of proposed intervention delivery [33].

## Results

### Phase 1: Understand the behaviour

#### Step 1: Define the problem in behavioural terms

The healthcare problem was the gap between current practice identified in the published literature [21, 23, 32] and international consensus guidelines to deliver sexual counselling to people with cardiovascular disease [17]. We identified increased delivery of sexual counselling as the behaviour to be targeted to address the problem.

#### Step 2: Select target behaviour

We did not identify a long list of candidate behaviours and select one as outlined in BCW guidance. Instead, international guidelines provided the target behaviour: the delivery of sexual counselling by healthcare professionals to people with cardiovascular disease.

#### Step 3: Specify the target behaviour

##### What is the behaviour that will be targeted for change?

The delivery of sexual counselling to people with cardiovascular disease was the behaviour targeted for change.

##### When and where is the behaviour performed?

Findings from the CHARMS baseline indicated that all stakeholders considered cardiac rehabilitation to be an appropriate setting for the delivery of sexual counselling [32]. Further specification of the target behaviour required an understanding of the format of Irish cardiac rehabilitation. There are 37 cardiac rehabilitation centres in Ireland which operate as units within a hospital. Cardiac rehabilitation is managed and delivered by a cardiac rehabilitation co-ordinator supported by a range of healthcare providers, including cardiologists, physiotherapists, nurses, occupational therapists, dieticians, pharmacists, psychologists and social workers.

There are four phases of cardiac rehabilitation, and each was considered as a potential implementation setting for the CHARMS intervention. Phase 1, hospitalisation following an acute cardiac event, was deemed unsuitable as patients may be too sick, and treatment too intensive, for the delivery of sexual counselling. Phase 2, the immediate post-discharge period, was also deemed not suitable as it is generally unstructured, variable in length, and input from health professionals may be minimal. Phase 3, structured exercise and education programmes delivered by cardiac rehabilitation staff in hospital outpatient units, was deemed to be suitable because (1) patients at this stage are engaged in a return to usual activities, including sex; (2) the intervention could be integrated into existing structured programmes; and (3) the intervention could be delivered by cardiac rehabilitation staff rather than by researchers, thus making the implementation sustainable. Phase 4, the on-going maintenance of lifestyle changes enacted in previous phases, was deemed to be unsuitable given the lack of structure and uncertain input from healthcare professionals.

International guidelines do not specify whether sexual counselling should be delivered on a one-to-one or group basis. However, as cardiac rehabilitation sessions are currently group-based, group delivery was chosen as the most feasible and sustainable delivery method. The potential spillover effects from group delivery was also promising as group discussion may facilitate the normalisation of sexual issues and empower patients to request one-to-one advice if needed.

##### Who performs the behaviour?

As cardiac rehabilitation can be delivered by different healthcare providers in different centres, the type of healthcare provider was not specified at this stage.

By answering the questions provided in BCW guidance, the target behaviour was specified as the provision of sexual counselling group sessions by cardiac rehabilitation healthcare staff to all patients aged 18 or older with cardiovascular disease during phase III cardiac rehabilitation in hospital centres in the Republic of Ireland.

#### Step 4: Identify what needs to change

By using the COM-B model to understand why sexual counselling is not currently being delivered by cardiac rehabilitation staff, we identified psychological capability, social opportunity, reflective motivation and automatic motivation as potentially important COM-B components to target. A summary of the identified barriers and associated COM-B components is provided in Table 1.

**Table 1:** Barriers to sexual counselling delivery, COM-B components, selected intervention functions, selected BCTs and BCT translation within the intervention

Barriers identified (source)	COM-B component	Selected intervention functions	Selected behaviour change techniques	Translation of behaviour change techniques within the intervention
Lack of knowledge and guidelines/information [23, 32] Low awareness among staff of sexual problems and cardiovascular disease [23]	Psychological Capability	Education	5.1 Information about health consequences	Provide information on clinical guidance about returning to sexual activity Provide information on improved health consequences for patients who receive sexual counselling
			5.6 Information about emotional consequences	Provide information on improved QOL/emotion for patients who receive sexual counselling
			6.3 Information about other’s approval	Discuss best practice guidelines developed by experts recommending sexual counselling
Lack of training in the provision of sexual counselling [23]	Psychological Capability	Training	4.1 Instruction on how to perform a behaviour	Provide manual and checklist of how to deliver group session Provide step-by-step guidance on how to address sexual concerns if raised
			6.1 Demonstration of behaviour	Show videos clips of good examples of HCPs interacting with patients who raise sexual concerns
			8.1 Behavioural practice/rehearsal	Role play exercises of interacting patients who raise sexual concerns
Perceptions among staff that the provision of sexual counselling to female patients is more difficult [23, 32]	Social opportunity	Enablement	1.2 Problem solving	Work with HCPs to identify potential problems related to gender and means to overcome barriers
			13.2 Framing/reframing	Suggest that provision of sexual counselling to women is particularly important given greater difficulties for women in discussing these issues
			15.1 Verbal persuasion about capability	Provide positive feedback in relation to role play performance and link with ability to provide sexual counselling in real-life settings
		Modelling	6.1 Demonstration of the behaviour	Show video clips of good communication around sexual problems with women
Perceptions among staff that issues related to patient culture, religion and ethnicity can make sexual counselling more difficult [23, 32]	Social opportunity	Enablement	1.2 Problem solving	Work with HCPs to identify potential problems related to culture, ethnicity and religion and means to overcome barriers
			13.2 Framing/reframing	Suggest that provision of sexual counselling to all is particularly important given greater cultural, religious and ethnic diversity
		Modelling	6.1 Demonstration of the behaviour	Show video clips of good communication around sexual problems with people from different ethnic groups
A sense of embarrassment and discomfort with sexual matters among staff, exacerbated by the older age of many patients [32] Staff members’ fear of offending patients should they broach the topic of sex and sexuality [32]	Automatic motivation	Modelling	6.1 Demonstration of the behaviour	Show video clips of interactions with patients that minimises potential offence and embarrassment
		Persuasion	5.1 Information about emotional consequences	Provide info on improved health outcomes for all patients
			6.3 Information about others’ approval	Provide information on patient’s expressed need for sexual counselling
			13.2 Framing/reframing	Reframe discussing sexual issues as meeting patients’ needs rather than causing offence
The perception among staff that patients do not expect staff to ask about sex [23] The perception among staff that giving permission is not a staff responsibility [23]	Reflective motivation	Education	6.3 Information about other’s approval	Give examples from the CHARMS baseline study showing how cardiac patients wanted and needed their healthcare providers to ask them about sex.
		Persuasion	6.2 Social comparisons	Show how sexual counselling is already part of routine cardiac rehabilitation in some centres in Ireland, and how it is integrated with rehabilitation in other countries
			9.1 Credible source	Provide information on the guidelines on sexual counselling during cardiac rehabilitation from the ESC and the AHA
Low confidence (among staff in the area of sexual counselling) [23]	Reflective motivation	Persuasion	15.1 Verbal persuasion about capability	The CHARMS educator will provide verbal support and reassurance throughout the training session, telling the staff members that they can successfully provide sexual counselling to their patients.
		Modelling	6.1 Demonstration of the behaviour	Show video clips depicting a cardiac rehabilitation staff member providing sexual counselling in a confident, assured manner.
Perceptions about the relationships between gender and age and sexuality [23, 32]	Reflective motivation	Persuasion	5.1 Information about health consequences	Provide info on improved health outcomes for all patients
			5.2 Salience of consequences	Provide case studies of positive consequences of providing sexual counselling including patients who vary by gender and age
			5.6 Information about emotional consequences	Provide info on improved quality of life and emotional outcomes for all patients
			6.3 Information about others’ approval	Provide information on patients’ expressed need for sexual counselling including patients who vary by gender and age
			9.1 Credible source	Ensure credibility of CHARMS educator and include expert video clips on benefits for all patients
			13.2 Framing/reframing	Reframe discussing sexual issues as meeting patients’ needs regardless of gender or age
Staff perceptions of patients’ lack of readiness and awareness with regard to sexual issues [23]	Reflective motivation	Persuasion	6.3 Information about others’ approval	Provide information on patients’ awareness of sexual issues and expressed need for sexual counselling during cardiac rehabilitation

##### Psychological capability

Both survey and focus group data indicated that staff felt they did not have the psychological capability to deliver sexual counselling and reported limited knowledge, awareness and information about sexual issues in cardiovascular disease and inadequate training in the skills required to deliver sexual counselling [23, 32].

##### Social opportunity

A number of social opportunity factors impacted on staff inclination to deliver sexual counselling to particular patient groups [32]. Older age was seen to be a barrier, with younger patients perceived to be more comfortable with discussions of sexuality. Male patients were seen to treat the discussion of sex in a jocular fashion; groups including women were seen to be more serious which may limit open discussion. A number of barriers particular to the delivery of sexual counselling to women were also discussed. Women were described as being unlikely to raise the topic of sexuality and to actively avoid cardiac rehabilitation sessions where sexuality would be discussed. Staff were also more reluctant to discuss sex with women as they believed older female patients had no experience or desire to discuss their sex lives with health professionals. Sexual dysfunction was also seen as more problematic and time-consuming for women, and to have fewer referral pathways available.

Social opportunity was also reflected in the issues around ethnicity, culture, language and religion identified as barriers to sexual counselling delivery in both the focus group and survey studies [23, 32]. Staff felt the discussion of sexual functioning might cause offence to some ethnic populations, or that staff from different cultural backgrounds may find the delivery of sexual counselling more difficult.

##### Reflective motivation

Perceived lack of patient readiness, not viewing assessment and counselling as a staff responsibility, and low staff confidence in their ability to deliver sexual counselling were identified as additional barriers across patient types [23].

##### Automatic motivation

The perceived difficulties in discussing sexual issues with patients also lead to negative emotional reactions to the delivery of sexual counselling that fall within the category of automatic motivation. In focus groups with cardiac staff, a sense of embarrassment and a fear of offending were two of the most frequently cited barriers to the discussion of sexuality [32] and discomfort with sexual matters was identified as a barrier in the staff survey [23].

### Phase 2: Identify intervention options

#### Step 5: Identify intervention functions

All nine intervention functions were potentially relevant to the COM-B components identified. The use of the APEASE criteria to determine the most relevant intervention functions within the context of phase 3 hospital cardiac rehabilitation in Ireland is shown in Additional file 1. Based on our application of the APEASE criteria, we selected education, enablement, modelling, persuasion and training as the most appropriate intervention functions.

#### Step 6: Identify policy categories

All seven policy categories were identified as potentially appropriate using BCW guidance, across the five intervention functions identified in step 5. Guidelines, regulation, legislation and service provision were common across at least four of the five included intervention functions. As regulation and legislation change were beyond the scope of our intervention, we identified guidelines and service provision as the two policy categories most relevant to the CHARMS intervention.

### Phase 3: Identify content and implementation options

#### Step 7: Identify behaviour change techniques

The initial long list of BCTs matched to intervention functions, and the initial selection of potential BCTs using the APEASE criteria, are shown in Additional file 2. From this list of potential BCTs, we selected 12 BCTs as the most appropriate following group discussion. Details of the selected BCTs linked to barriers and intervention functions are shown in Table 1.

#### Step 8: Identify mode of delivery

For mode of delivery, the items included in the TIDieR checklist provided a guide to the specification of the planned delivery of intervention content. The completed TIDieR checklist is shown in Additional file 3.

### The CHARMS implementation intervention

The CHARMS implementation intervention consists of the delivery of the 12 BCTs to all cardiac rehabilitation healthcare providers as a face-to-face group intervention lasting approximately 2 h. The translation of chosen BCTs into intervention content is shown in Table 1. The intervention will be delivered by a CHARMS educator. Grimshaw et al. (2011) highlight the importance of credibility and suggest that the most appropriate person to deliver an implementation intervention varies according to the target audience [34]. The four criteria set for the CHARMS educator were as follows:

1. Be from a health profession currently represented within usual cardiac rehabilitation
2. Have experience with dealing with sexual issues among patients with cardiovascular disease
3. Be experienced in cardiac rehabilitation
4. Be able to advise on acceptability or feasibility issues with the intervention and study protocol

The CHARMS intervention delivered by the CHARMS educator will support staff to deliver a 30-min group-based sexual counselling session to patients within the cardiac rehabilitation programme. Here again, a system-level consideration was important as not all cardiac rehabilitation centres work in exactly the same way. For example, the number of staff and the length of the rehabilitation programme can vary, as can the number of patient groups running at any one time. Therefore, sexual counselling delivery needs to be integrated into the rehabilitation programme at each centre in a flexible way. The CHARMS educator will discuss, agree and document with staff details of the integration of group sexual counselling into the routine cardiac rehabilitation programme.

Staff will be provided with an intervention manual (with content linked to BCTs defined in the BCTTv1) which includes additional detail to support the intervention content delivered by the CHARMS educator and step-by-step guidance on the delivery of sexual counselling at the patient level. Staff will also be provided with an information booklet for patients containing information on the resumption of sexual activity after a cardiac event and suggestions about how to deal with problems when they arise. Partners of patients will be invited to attend the session in line with international guidance [17, 18]. Study posters will also be displayed in the participating cardiac rehabilitation centres to reassure patients that a return to sexual activity after a cardiac event is normal and to encourage patients to seek advice from staff if they have a sexual problem.

## Discussion

This paper describes the methods used to systematically develop the multi-level CHARMS implementation intervention to increase uptake of sexual counselling delivery guidelines by hospital cardiac rehabilitation staff in Ireland. The paper addresses a clear evidence-practice gap in the delivery of sexual counselling and describes a transparent intervention development process informed by focus group and survey data from cardiac rehabilitation staff and patients. The paper extends behavioural science methodology by using the BCW to develop an intervention in an under-researched and sensitive area of healthcare provision, the delivery of sexual counselling during cardiac rehabilitation. Our use of a systematic approach, identification of mechanisms of action and description of intervention content using standard terminology will allow the CHARMS intervention, whether effective or not, to contribute to the cumulative science of implementation intervention development. The paper also provides a worked example and a reflection on the application of the BCW to implementation intervention development, which may be useful for other researchers.

### Reflections on the BCW approach

We found that the BCW provided a useful framework to move from our initial exploratory research in the area of sexuality and health services for people with cardiovascular disease towards intervention formulation, particularly at the provider level. Michie and colleagues [28] suggest that the major strength of the BCW approach is that it encourages intervention designers to consider the full range of options and to choose the most promising through a systematic evaluation of theory and evidence. Our experience supports this suggestion, and the BCW approach allowed us to identify all potentially relevant intervention functions and BCTs and to select the most appropriate. The use of the BCT taxonomy allowed us to specify content in detail, using agreed labels and definitions. We found the use of standardised terminology and pre-determined phases of the development process to be invaluable in facilitating discussion within the research team.

Guidance for taking a systematic approach to designing interventions is also provided by frameworks other than the BCW. Intervention mapping, for example, outlines an alternative approach to developing theory- and evidence-based health promotion programmes [35]. One of the strengths we identified in our use of the BCW is the simplicity and coherence of the COM-B model at the core. A systematic review of the extent of use of theory in implementation research found that only 53 of 235 studies (22.5 %) had employed theories [36]. A traditional issue with theory, particularly when working in a multi-disciplinary team, is the difficulty in selecting the most appropriate theory from a potentially long list of options [37]. COM-B provides a simplified framework suitable for application to behaviour in any setting.

We found some of the initial steps to define and select the target behaviour to be less fundamental to the development of our intervention. Although we have outlined all of the steps included in BCW guidance in the current study, in reality, international guidelines provided the target behaviour: the delivery of sexual counselling by healthcare professionals to people with cardiovascular disease. The target behaviour may be pre-specified in other implementation interventions designed to support uptake of guidelines into practice or be decided a priori in funding applications to support intervention development.

We identified some potential ambiguities in the use of the COM-B model to identify what needs to change to alter behaviour, as exploratory data could potentially be coded in different ways. For example, barriers identified by staff around patient lack of readiness could be coded as social opportunity if seen as a reflection of the reality of Irish social and cultural norms. However, studies with patients contradict staff perceptions, as patients report a desire for sexual issues to be raised by their healthcare professional [32]. Accordingly, perceived lack of patient readiness may be best understood as a staff member’s personal evaluations of the situation, rather than reality, and be coded as reflective motivation within the COM-B model. Multiple perspectives are an inherent characteristic of multi-level implementation interventions, and more examples of how approaches such as the BCW can be applied across patient, provider, system and environmental levels of change would be helpful in navigating the complexities of real-world implementation.

Further guidance on the translation of BCTs into intervention content would also be helpful. Although this is covered to some extent in BCW guidance under mode of delivery in phase 3 [28], a more explicit thorough approach to the specification of proposed intervention delivery should be encouraged. We found the TIDieR checklist [33] to be a useful tool to help specify details of proposed intervention delivery including by who, how and where the intervention would be delivered,and recommend its use as an additional phase in the BCW approach.

The BCW includes seven broad policy categories that can be used to leverage behaviour change. In line with other published examples, we found the selection of policy level categories less well defined and practical than the other BCW steps [26, 30]. This recurring difficulty in identifying policy categories may reflect an issue with how policy is currently represented as part of the BCW approach. The identification of policy categories is currently described as step 6 of the BCW process, between the identification of intervention functions and the identification of BCTs. In practice, we found the identification of intervention functions to flow logically to the selection of BCTs, without the identification of policy categories. For implementation interventions, the policy level may be better represented as a broader over-arching aspect of the process, more in line with the consideration of the environmental level of implementation than a discreet step in intervention development to change healthcare professional behaviour.

### Limitations

In the current study, three members of the core research team were involved in moving through the phases outlined in the BCW guidance and our multi-disciplinary steering committee approved the final intervention content. Previous published examples have organised expert workshops or multi-disciplinary consensus meetings structured around the BCW [26, 38]. The lack of healthcare professional and patient input into our BCW intervention development process is a limitation of the current study. The CHARMS intervention is currently being pilot tested in two cardiac rehabilitation centres and the views of healthcare professionals, and patients/partners, on the feasibility and acceptability of the CHARMS intervention will be collected via questionnaires and interviews and be used for the further refinement of the intervention [39]. The CHARMS educator will also feed into the intervention refinement process. Although a limitation, the current study demonstrates that the BCW can be used to support systematic intervention development even when resources to support larger expert consensus consultation are not available. The time and funding investment required to take a systematic approach to intervention development has been previously noted [26, 30, 31], and the current study provides an example of the use of the BCW approach on a slightly less resource-intensive scale.

We used the COM-B model to understand why sexual counselling is not currently being carried out, rather than the more detailed Theoretical Domains Framework [40] which has previously been used in the development of theory-informed implementation interventions using the BCW approach [31]. One previous study compared the use of both COM-B and the Theoretical Domains Framework and concluded that similar associations between identified barriers, intervention functions and BCTs were found using both methods [26]. We found that the COM-B provided a sufficient framework for developing our intervention but that at times referring to the more detailed constructs included in the Theoretical Domains Framework, which have been linked to the COM-B components, was useful in clarifying uncertainty when moving through the BCW process.

### Implications for research and practice

The current study highlights the importance of preliminary research with key stakeholders in the design of implementation interventions. The BCW approach provides a framework for intervention development, but requires the use of subjective judgement at every phase. Data collected as part of the CHARMS baseline were vital to inform our decisions throughout the intervention development and provided a consistent reminder of the reality of the Irish cardiac rehabilitation healthcare context. Exploratory work with stakeholders may be particularly important for sensitive topics, such as sexuality, to identify potential emotional and social barriers to implementation.

Use of the COM-B model, and linking COM-B components to intervention functions and specific BCTs, allowed us to explicitly outline the proposed mechanisms of action of the CHARMS intervention. Investigating these hypothesised mechanisms of action, and whether included BCTs are effective, requires a rigorous evaluation of the intervention. A detailed evaluation, including an economic evaluation and assessment of intervention feasibility and fidelity, is planned for the CHARMS intervention. Provider and patient level outcomes and staff capability, opportunity and motivation for providing sexual counselling in cardiac rehabilitation pre- and post-intervention will be measured. Further detail on the planned intervention evaluation is provided in the protocol for the CHARMS implementation intervention pilot study [39].

Although the benefits of systematic intervention development are generally accepted [41], the question as to whether using the BCW and other systematic approaches increases the effectiveness of interventions has not yet been addressed. With the recent publication of a number of examples of interventions developed using the BCW approach [26, 30, 38], the time may now be ripe to explore the acceptability, feasibility and effectiveness of interventions developed using the BCW relative to other interventions.

The environmental level of implementation was not addressed in the current study. If, in a future randomised controlled trial, we demonstrate that the CHARMS intervention is effective, the widespread implementation of the CHARMS intervention would require further development at an environmental level. The Irish Association of Cardiac Rehabilitation (IACR) is a multi-disciplinary group supported by the Irish Heart Foundation, a national heart disease and stroke charity. The IACR aims to increase awareness and understanding of cardiac rehabilitation in Ireland, improve the standard of professional education, promote an evidence-based approach to client care and facilitate communication and support between cardiac rehabilitation multi-disciplinary professionals. If the CHARMS intervention is shown to be effective and feasible, the research team will work with the IACR to establish how best to incorporate the intervention at an environmental level into national cardiovascular rehabilitation policy and training frameworks.

## Conclusions

The BCW approach provides a systematic, explicit and pragmatic framework for implementation intervention development. Additional guidance on the translation of BCTs into intervention content and a re-consideration of the representation of policy within the BCW may increase the usefulness of the approach. Additional examples of how the BCW can be applied across environmental, system, provider and patient levels would also be helpful to inform future development of complex implementation interventions.

## Additional files

Additional file 1: Step 5: Use of APEASE criteria to identify potentially relevant intervention functions. (DOCX 17 kb)
Additional file 2: Step 7: Use of APEASE criteria to identify potentially relevant BCTs. (DOCX 21 kb)
Additional file 3: Step 7: Completed TIDieR checklist. (DOCX 30 kb)
