# Supplementary material for: Implementing international sexual counselling guidelines in hospital cardiac rehabilitation: development of the CHARMS intervention using the Behaviour Change Wheel
Source: Implement Sci. 2016 Oct 10;11:134. doi: 10.1186/s13012-016-0493-4 (PMC5057276; doi:10.1186/s13012-016-0493-4)
Supplement: Additional file 1: — Step 5: Use of APEASE criteria to identify potentially relevant intervention functions. (DOCX 17 kb) [file 13012_2016_493_MOESM1_ESM.docx]

**Supplementary Material 1**

**Step 5: Use of APEASE criteria to identify potentially relevant intervention functions**

| **BCW Intervention Functions** | **Affordability** | **Practicability** | **Effectiveness and cost effectiveness** | **Acceptability** | **Side effects/ safety** | **Equity** | **Comments** | **Decision**  **Yes/No** |
| --- | --- | --- | --- | --- | --- | --- | --- | --- |
| Coercion | ✓ | 🗶 | ✓ | 🗶 | 🗶 | ✓ | Creating an expectation of punishment or cost was not thought to be acceptable for CR staff that would be expected to provide care to patients in a fairly autonomous way. It was also judged that CR staff would not accept the imposition of punishments or costs, and side-effects might have included outright rejection of the CHARMS intervention. | No |
| Education | ✓ | ✓ | ✓ | ✓ | ✓ | ✓ | Education was judged to meet all of the APEASE criteria:  - Affordability: it is covered within budgetary allocations  - Practicability: it can be delivered as a staff training module  - Effectiveness: this is uncertain, but judged to be worth evaluating as part of the pilot study  - Acceptability: CR staff would welcome relevant education and training  - Side-effects: risk of unwanted side-effects was judged to be minimal  - Equity: no negative impact | Yes |
| Enablement | ✓ | ✓ | ✓ | ✓ | ✓ | ✓ | Enablement was judged to meet all of the APEASE criteria:  - Affordability: budgetary provision is already in place for a patient intervention manual, a patient booklet, and posters.  - Practicability: providing these means of enablement is part of the current project plan  - Acceptability: staff have frequently commented on the need for materials that would enable their provision of sexuality-related care.  - Effectiveness: this is uncertain, but judged to be worth evaluating as part of the pilot study  - Side-effects: risk of unwanted side-effects was judged to be minimal  - Equity: no negative impact | Yes |

| Environmental Restructuring | 🗶 | 🗶 | ✓ | 🗶 | ✓ | ✓ | Phase III cardiac rehabilitation already occurs in a structured physical and social environment. Restructuring would require budgetary resources quite beyond those available, and so was deemed to be both unaffordable and impracticable. Additionally, it was judged unlikely that CR management and staff would find attempts at environmental restructuring acceptable, as that could be interpreted as a radical intrusion on their work practices. | No |
| --- | --- | --- | --- | --- | --- | --- | --- | --- |
| Incentivisation | ✓ | 🗶 | ✓ | 🗶 | 🗶 | ✓ | Utilising an incentivisation function was not judged to be practicable due to budgetary constraints. Nor was it thought to be acceptable given the sensitive nature of the intervention, and it could have unwanted side-effects in terms of creating tension among staff. | No |
| Modelling | ✓ | ✓ | ✓ | ✓ | ✓ | ✓ | Modelling was judged to meet all of the APEASE criteria:  - Affordability: This would not impact on the budget  - Practicability: A nurse trainer will provide opportunities for modelling during the staff intervention, and trained staff will model the targeted behaviours for others  - Effectiveness: this is uncertain, but judged to be worth evaluating as part of the pilot study  - Acceptability: given the uncertainty displayed by staff in relation to sexual matters, it is thought the provision of models for the targeted behaviours would be highly acceptable.  - Side-effects: risk of unwanted side-effects was judged to be minimal  - Equity: no negative impact | Yes |
| Persuasion | ✓ | ✓ | ✓ | ✓ | ✓ | ✓ | Persuasion was judged to meet all of the APEASE criteria:  - Affordability: covered within budgetary allocations  - Practicability: during staff training, qualitative data from CR patients can be used to induce positive emotions about provision of sexuality-related care  Effectiveness: this is uncertain, but judged to be worth evaluating as part of the pilot study  Acceptability: inducing positive emotions about this matter was deemed to be acceptable.  - Side-effects: risk of unwanted side-effects was judged to be minimal  - Equity: no negative impact | Yes |
| Restriction | ✓ | 🗶 | ✓ | 🗶 | 🗶 | ✓ | It was not thought practical to attempt restriction of staff behaviour when, again, they would be expected to provide patient care autonomously. Restriction was not thought to be acceptable as it would ‘limit agency on the part of the target group’, and side-effects of that might include outright rejection of the CHARMS intervention. | No |
| Training | ✓ | ✓ | ✓ | ✓ | ✓ | ✓ | Training was judged to meet all of the APEASE criteria:  - Affordability: it is covered within budgetary allocations  - Practicability: it can be delivered as a staff training module  - Effectiveness: this is uncertain, but judged to be worth evaluating as part of the pilot study  - Acceptability: CR staff would welcome relevant education and training  - Side-effects: risk of unwanted side-effects was judged to be minimal  - Equity: no negative impact | Yes |
