# Supplementary material for: Implementing international sexual counselling guidelines in hospital cardiac rehabilitation: development of the CHARMS intervention using the Behaviour Change Wheel
Source: Implement Sci. 2016 Oct 10;11:134. doi: 10.1186/s13012-016-0493-4 (PMC5057276; doi:10.1186/s13012-016-0493-4)
Supplement: Additional file 2: — Step 7: Use of APEASE criteria to identify potentially relevant BCTs. (DOCX 21 kb) [file 13012_2016_493_MOESM2_ESM.docx]

**Supplementary Material 2**

**Step 7: Use of APEASE criteria to identify potentially relevant BCTs**

| **Intervention Function** | **Relevant BCTs identified from Michie et al 2014 [**[**26**](#_ENREF_26)**]** | **Does the BCT meet the APEASE criteria in the context?** |
| --- | --- | --- |
| Education | 2. Feedback and Monitoring |  |
|  | 2.2 Feedback on behaviour | Not really practical as all relevant behaviour will not be monitored-PM  Probably not possible as only HCPs will know if they have delivered counselling-JMS |
|  | 2.3 Self-monitoring of behaviour | Data collection rather than behaviour change strategy-PM  Might it be useful to include-JMS  Decision: No |
|  | 2.4 Self-monitoring of outcomes of behaviour | Part of data collection rather than behaviour change strategy. |
|  | 2.6 Biofeedback | Not relevant |
|  | 2.7 Feedback on outcomes of the behaviour | Not practical |
|  | 4. Shaping Knowledge |  |
|  | 4.2 Information about antecedents | Not relevant-PM  Could this be included in Education-JMS  Decision: No |
|  | 4.3 Re-attribution | Not acceptable to question attributions made by HCPs |
|  | 4.4 Behavioural experiments | Not practical |
|  | 5. Natural Consequences |  |
|  | 5.1 Information about health consequences | Yes |
|  | 5.3 Information about social and environmental consequences | Not relevant-PM  Could this maybe be creating a more open environment for patients? JMS  Decision: No |
|  | 5.6 Information about emotional consequences | Yes |
|  | 6. Comparison of Behaviour |  |
|  | 6.3 Information about other's approval | Yes |
|  | 7. Associations |  |
|  | 7.1 Prompts/cues | Not relevant-PM  Might the tick box on the form be a possible prompt/cue?-JMS  Decision: No |
|  | 7.2 Cue signalling reward | Not relevant |
|  | 7.6 Satiation | Not relevant |
| Persuasion | 2. Feedback and Monitoring |  |
|  | 2.2 Feedback on behaviour | Not really practical as all relevant behaviour will not be monitored-PM  See above-JMS |
|  | 2.6 Biofeedback | Not relevant |
|  | 2.7 Feedback on the outcomes of behaviour | Not practical |
|  | 4. Shaping Knowledge |  |
|  | 4.3 Re-attribution | Not acceptable to question attributions made by HCPs |
|  | 5. Natural Consequences |  |
|  | 5.1 Information about health consequences | Yes |
|  | 5.2 Salience of consequences | Yes (perhaps could do this using quotes from qualitative work?)-PM  Yes-JMS |
|  | 5.3 Information about social and environmental consequences | Not relevant-PM  See above-JMS  Decision: No |
|  | 5.6 Information about emotional consequences | Yes |
|  | 6. Comparison of behaviour |  |
|  | 6.2 Social comparison | Yes (comparison to other clinics, other countries, etc.) |
|  | 6.3 Information about others' approval | Yes |
|  | 9. Comparison of Outcomes |  |
|  | 9.1 Credible source | Yes (international guidelines available)-PM  Yes (maybe a video could be included in the training as a credible source)- JMS |
|  | 13. Identity |  |
|  | 13.1 Identification of self as role model | Not practical/relevant in this context-PM  Might this be relevant to the coordinator at each centre?-JMS  Decision: No |
|  | 13.2 Framing/reframing | Yes - reframe sexual counselling as reducing distress rather than causing distress |
|  | 13.5 Identity associated with changed behaviour | Not relevant |
|  | 15. Self-belief |  |
|  | 15.1 Verbal persuasion about capability | Yes |
|  | 15.3 Focus on past success | Unlikely to be applicable to target HCPs |
| Training | 2. Feedback and Monitoring |  |
|  | 2.2 Feedback on behaviour | Not really practical as all relevant behaviour will not be monitored-PM  See above-JMS |
|  | 2.3 Self-monitoring of behaviour | Part of data collection-PM  See above-JMS  Decision: No |
|  | 2.4 Self-monitoring of outcomes of behaviour | Part of data collection rather than behaviour change strategy. |
|  | 2.6 Biofeedback | Not relevant |
|  | 2.7 Feedback on outcomes of behaviour | Not practical |
|  | 4. Shaping Knowledge |  |
|  | 4.1 Instruction on how to perform a behaviour | Yes |
|  | 4.4 Behavioural experiments | Not practical |
|  | 6.0 Comparison of Behaviour |  |
|  | 6.1 Demonstration of behaviour | Yes |
|  | 8. Repetition and substitution |  |
|  | 8.1 Behavioural practice/rehearsal | Yes |
|  | 8.3 Habit formation | Not practical to encourage habit formation-PM  Maybe this could be included? JMS  Decision: No |
|  | 8.4 Habit reversal | Not practical to think of behaviour as a habit |
|  | 8.7 Graded tasks | Not relevant |
|  | 10. Reward and threat |  |
|  | 10.9 Self-reward | Not acceptable for highly trained HCPs |
|  | 15. Regulation |  |
|  | 15.2 Mental rehearsal of successful performance | Not practical. Behaviour too complex to meaningfully imagine it. |
|  | 15.4 Self-talk | Not acceptable for highly trained HCPs |
| Modelling | 6. Comparison of the Behaviour |  |
|  | 6.1 Demonstration of the behaviour | Yes |
| Enablement | 1. Goals and Planning |  |
|  | 1.1 Goal setting (behaviour) | I doubt that HCPs would find this acceptable-PM  Maybe-JMS  Decision: No |
|  | 1.2 Problem solving | Not practical (already done in previous research)-PM  I think this could be important-JMS  Decision: Yes |
|  | 1.3 Goal setting (outcome) | I doubt that HCPs would find this acceptable-PM |
|  | 1.4 Action planning | Yes |
|  | 1.5 Review behaviour goals | Not acceptable (as goal setting not acceptable IMO)-PM  Maybe-JMS  Decision: No |
|  | 1.6 Discrepancy between current behaviour and goal | Won’t have record of current behaviour |
|  | 1.7 Review outcome goals | Not engaging in goal setting |
|  | 1.8 Behavioural contract | Not acceptable |
|  | 1.9 Commitment | Not acceptable  Maybe-JMS  Decision: No |
|  | 2. Feedback and Monitoring |  |
|  | 2.3 Self-monitoring of behaviour | See above  Decision: No |
|  | 2.4 Self-monitoring of outcomes of behaviour | Part of data collection |
|  | 3. Social Support |  |
|  | 3.1 Social support (unspecified) | Not practical |
|  | 3.2 Social support (practical) | Yes (practical support can be provided by PM via email and freephone number)-PM  Could this be through coordinator? JMS  Decision: Yes |
|  | 3.3 Social support (emotional) | Not practical |
|  | 4. Shaping Knowledge |  |
|  | 4.4 Behavioural experiments | Not practical |
|  | 5. Natural Consequences |  |
|  | 5.2 Salience of consequences | Yes (perhaps could do this using quotes from qualitative work?) |
|  | 5.4 Monitoring of emotional consequences | Not acceptable/practical |
|  | 5.5 Anticipated regret | Not relevant |
|  | 8. Repetition and substitution |  |
|  | 8.2 Behaviour substitution | Not relevant |
|  | 8.5 Overcorrection | Not relevant |
|  | 8.6 Generalisation of a target behaviour | Not relevant |
|  | 8.7 Graded tasks | Not relevant |
|  | 9. Comparison of outcomes |  |
|  | 9.2 Pros and cons | Not practical (already established in previous research)-PM  Maybe-JMS  Decision: No |
|  | 9.3 Comparative imagining of future outcomes | Not practical |
|  | 10. Reward and threat |  |
|  | 10.9 Self-reward | Not acceptable |
|  | 11. Regulation |  |
|  | 11.1 Pharmacological support | Not relevant |
|  | 11.2 Reduce negative emotions | Yes (can make use of literature about sexuality and the older person)-PM  Is this to reduce the negative emotions of the HCP?-JMS  Decision: Yes |
|  | 11.3 Conserve mental resources | Not relevant |
|  | 12. Antecedents |  |
|  | 12.1 Restructuring the physical environment | Not practical/acceptable-PM  Might this include posters/booklets?-JMS  Decision: No |
|  | 12.2 Restructuring the social environment | Not practical |
|  | 12.3 Avoidance/reducing exposure to cues for the behaviour | Not relevant |
|  | 12.4 Distraction | Not relevant |
|  | 12.5 Adding objects to the environment | Not relevant-PM  Might this include posters/booklets?-JMS  Decision: No |
|  | 12.6 Body changes | Not relevant |
|  | 13. Identity |  |
|  | 13.1 Identification of self as role model | See above  Decision: No |
|  | 13.2 Framing/reframing | Yes - reframe sexual counselling as reducing distress rather than causing distress |
|  | 13.3 Incompatible beliefs | Yes |
|  | 13.4 Valued self-identity | Not acceptable to HCPs |
|  | 13.5 Identity associated with changed behaviour | Not acceptable to HCPs |
|  | 15. Self-belief |  |
|  | 15.1 Verbal persuasion about capability | Yes |
|  | 15.2 Mental rehearsal of successful performance | Not practical. Behaviour too complex to meaningfully imagine it. |
|  | 15.3 Focus on past success | Not practical (unlikely to have past successes to draw on) |
|  | 15.4 Self-talk | Not acceptable to HCP’s! |
|  | 16. Covert learning |  |
|  | 16.1 Imaginary punishment | Not acceptable |
|  | 16.2 Imaginary reward | Yes (although I’m doubtful about this)-PM  I’m a little doubtful too!-JMS  Decision: Yes |
|  | 16.3 Vicarious consequences | Not practical as CR staff would not have opportunity to observe in this way. |
