# Supplementary material for: Implementing international sexual counselling guidelines in hospital cardiac rehabilitation: development of the CHARMS intervention using the Behaviour Change Wheel
Source: Implement Sci. 2016 Oct 10;11:134. doi: 10.1186/s13012-016-0493-4 (PMC5057276; doi:10.1186/s13012-016-0493-4)
Supplement: Additional file 3: — Step 7: Completed TIDieR checklist. (DOCX 30 kb) [file 13012_2016_493_MOESM3_ESM.docx]

**Supplementary Material 3**

**Step 7: Completed TIDieR checklist**


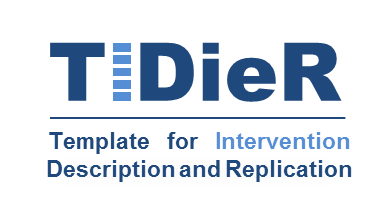


**The TIDieR (Template for Intervention Description and Replication) Checklist**

Information to include when describing an intervention and the location of the information

| **Item number** | **Item** |  |
| --- | --- | --- |
|  |  |  |
|  | **BRIEF NAME** |  |
| **1.** | Provide the name or a phrase that describes the intervention. | Cardiac Health and Relationship Management and Sexuality (CHARMS) sexual counselling intervention. |
|  | **WHY** |  |
| **2.** | Describe any rationale, theory, or goal of the elements essential to the intervention. | The CHARMS intervention was developed using the Behaviour Change Wheel approach and aims to improve implementation of guidelines by increasing sexual counselling delivery by healthcare providers in hospital-based cardiac rehabilitation. |
|  | **WHAT** |  |
| **3.** | Materials: Describe any physical or informational materials used in the intervention, including those provided to participants or used in intervention delivery or in training of intervention providers. Provide information on where the materials can be accessed (e.g. online appendix, URL). | The CHARMS intervention includes:  (1) The CHARMS staff intervention delivered by a CHARMS educator using a manual (development described in the current paper)  (2) The CHARMS patient intervention delivered by a member of the cardiac rehabilitation staff using a manual  (3) Patient booklet  (4) An awareness raising poster  Further details are provided in the CHARMS pilot protocol paper. |
| **4.** | Procedures: Describe each of the procedures, activities, and/or processes used in the intervention, including any enabling or support activities. | The CHARMS staff intervention consists of the delivery of 12 BCTs to cardiac rehabilitation healthcare providers by a CHARMS Educator. The CHARMS staff intervention will support staff to deliver the CHARMS patient intervention, a sexual counselling session, to patients during cardiac rehabilitation.  A patient information booklet will be provided to patients with information on the resumption of sexual activity after a cardiac event.  Study posters will be displayed in hospitals to reassure patients that a return to sexual activity after a cardiac event is normal and to encourage patients to seek advice from staff if they have a sexual problem. |
|  | **WHO PROVIDED** |  |
| **5.** | For each category of intervention provider (e.g. psychologist, nursing assistant), describe their expertise, background and any specific training given. | Criteria for the CHARMS Educator:   1. Be from a health profession currently represented within usual cardiac rehabilitation 2. Have experience with dealing with sexual issues among patients with cardiovascular disease. 3. Be experienced in cardiac rehabilitation 4. Be able to advise on acceptability or feasibility issues with the intervention and study protocol.   The CHARMS Educator will be briefed by the research team and provided with the staff intervention manual and slides for presentation to staff.  All staff at the participating cardiac rehabilitation centres will be invited to attend the CHARMS staff intervention and will work with the CHARMS educator to identify a staff member to deliver the patient intervention. |
|  | **HOW** |  |
| **6.** | Describe the modes of delivery (e.g. face-to-face or by some other mechanism, such as internet or telephone) of the intervention and whether it was provided individually or in a group. | The CHARMS staff intervention will be delivered in a face to face group session by a CHARMS Educator using a pre-developed manual. The CHARMS patient intervention will be delivered in a face to face group session by a cardiac rehabilitation staff member using a pre-developed manual. |
|  | **WHERE** |  |
| **7.** | Describe the type(s) of location(s) where the intervention occurred, including any necessary infrastructure or relevant features. | The CHARMS staff intervention will be delivered at cardiac rehabilitation hospital centres. The CHARMS patient intervention will be embedded into Phase III cardiac rehabilitation at hospital centres as an additional group-based session. |
|  | **WHEN and HOW MUCH** |  |
| **8.** | Describe the number of times the intervention was delivered and over what period of time including the number of sessions, their schedule, and their duration, intensity or dose. | The CHARMS staff intervention will be delivered once and last for approximately 2 hours. The CHARMS patient intervention will be delivered once during Phase III cardiac rehabilitation and last approximately 30 minutes. |
|  | **TAILORING** | N/A (intervention not yet delivered). |
| **9.** | If the intervention was planned to be personalised, titrated or adapted, then describe what, why, when, and how. |  |
|  | **MODIFICATIONS** |  |
| **10.^ǂ^** | If the intervention was modified during the course of the study, describe the changes (what, why, when, and how). | N/A (intervention not yet delivered). |
|  | **HOW WELL** |  |
| **11.** | Planned: If intervention adherence or fidelity was assessed, describe how and by whom, and if any strategies were used to maintain or improve fidelity, describe them. | Fidelity of intervention delivery will be assessed using trainer self-reports, researcher-coded audio recordings and exit interviews.  Further details are provided in the CHARMS pilot protocol paper. |
| **12.^ǂ^** | Actual: If intervention adherence or fidelity was assessed, describe the extent to which the intervention was delivered as planned. | N/A (intervention not yet delivered). |

** **Authors** - use N/A if an item is not applicable for the intervention being described. **Reviewers** – use ‘?’ if information about the element is not reported/not sufficiently reported.

† If the information is not provided in the primary paper, give details of where this information is available. This may include locations such as a published protocol or other published papers (provide citation details) or a website (provide the URL).

ǂ If completing the TIDieR checklist for a protocol, these items are not relevant to the protocol and cannot be described until the study is complete.
* We strongly recommend using this checklist in conjunction with the TIDieR guide (see *BMJ* 2014;348:g1687) which contains an explanation and elaboration for each item.

* The focus of TIDieR is on reporting details of the intervention elements (and where relevant, comparison elements) of a study. Other elements and methodological features of studies are covered by other reporting statements and checklists and have not been duplicated as part of the TIDieR checklist. When a **randomised trial** is being reported, the TIDieR checklist should be used in conjunction with the CONSORT statement (see [www.consort-statement.org](http://www.consort-statement.org)) as an extension of **Item 5 of the CONSORT 2010 Statement.** When a **clinical trial** **protocol** is being reported, the TIDieR checklist should be used in conjunction with the SPIRIT statement as an extension of **Item 11 of the SPIRIT 2013 Statement** (see [www.spirit-statement.org](http://www.spirit-statement.org)). For alternate study designs, TIDieR can be used in conjunction with the appropriate checklist for that study design (see [www.equator-network.org](http://www.equator-network.org)).
